# A Modified Guideline for High-Fidelity Patient Simulation to Improve Student Satisfaction and Self-Confidence in Learning: A Mixed Study

**DOI:** 10.3390/nursrep13030090

**Published:** 2023-07-28

**Authors:** Florence M. F. Wong, David C. N. Wong

**Affiliations:** 1 School of Nursing, Tung Wah College, Kowloon, Hong Kong SAR 999078, China; 2 Research Office, Tung Wah College, Kowloon, Hong Kong SAR 999078, China; shallwex@yahoo.com.hk

**Keywords:** high-fidelity, simulation, satisfaction, confidence, learning, nursing education

## Abstract

The coronaviral pandemic has led to a shift in traditional teaching methods to more innovative approaches, such as high-fidelity patient simulation (HFPS), which can improve students’ clinical judgment and decision making for quality patient care. A modified guideline was introduced to enhance students’ satisfaction and self-confidence in learning through HFPS. The study involved 189 baccalaureate nursing students, with 92 in the intervention group and 97 in the control group. The intervention group received the modified HFPS guideline, while the control group received standard treatment with basic instruction. After the HFPS debriefing session, students provided narrative feedback on their learning experiences. The quantitative results showed that students in the intervention group reported a significant improvement in satisfaction and self-confidence in learning compared to the control group. The modified HFPS guideline provided clear guidance for students to learn and apply knowledge and skills more effectively, leading to increased engagement during interactive simulation sessions. The results suggest that the HFPS guideline should be added to the curriculum to enhance students’ satisfaction and self-confidence in learning, even for junior students. After the pandemic, innovative teaching methods, such as HFPS, can be necessary and beneficial for healthcare professional training.

## 1. Introduction

The COVID-19 pandemic has had a significant impact on students’ learning attitudes and behaviors, necessitating the use of innovative methods to encourage and enhance learning. It has presented new challenges for nurses, who now face more complex and immediate clinical situations. In addition to the advanced technology utilized in healthcare services, nurses are expected to take on greater accountability for managing complex clinical judgments and decisions. These higher expectations are necessary to ensure effective and appropriate patient care [1,2]. However, nurses often encounter challenges in making immediate clinical judgments and decisions [3]. As a result, there is a growing need for innovative, cost-effective, and high-quality training programs aimed at enhancing nursing competence and ultimately benefiting both patient care and healthcare services.

High-fidelity patient simulation (HFPS) is an advanced technology-based method widely used in professional training, including healthcare services. It has been shown to effectively improve knowledge acquisition and skill performance, enhancing clinical competence [4,5,6]. Nursing education includes theoretical knowledge, psychomotor skills training, and scenario-based nursing practice to improve competence in safe and appropriate practice [7,8]. Students can perform their learned knowledge and skills to foster clinical competence and ensure patient safety in a controlled and risk-free environment [9,10,11,12]. Students learn their roles and responsibilities in HFPS situations, discover their strengths and weaknesses [12,13], and develop motivation for lifelong learning and collaborative teamwork [13,14,15,16]. Students can interact and collaborate with their peers to exchange their learning experiences, enhancing their competence in nursing practice and teamwork skills in the HFPS. Therefore, HFPS acts as an important innovative teaching–learning method to foster students’ ability in clinical judgment and decision making. However, students’ satisfaction and confidence in learning through HFPS directly affect their motivation and engagement [17].

To address this, HFPS provides simulated patient training scenarios in clinical settings, allowing students to integrate their knowledge and psychomotor skills [18,19]. With the application of HFPS in the last decades, students have learned more effectively when they engage in this innovative learning activity [19,20,21]. Studies have shown that HFPS improves engagement, learning achievement, satisfaction, and confidence levels among students [22,23]. Therefore, it is important to increase their willingness and interest in learning. Students’ satisfaction and their confidence in learning are essential elements, and they are intertwined. The more satisfaction students have, the more confidence they have to motivate themselves to undertake thinking and learning challenges [5,11]. HFPS is a multifaceted learning approach that necessitates students to engage in role playing and maximize their knowledge acquisition throughout the entire HFPS process [24]. With the growing popularity of simulations across various educational levels, different academic institutions have developed their unique simulation guidelines. HFPS is recognised as a motivating, secure, and cost-effective approach that not only provides students with hardware simulation devices but also incorporates engaging and instructive learning materials that are tailored to a specific learning environment. As a result, a well-designed HFPS is imperative in facilitating students’ involvement in this high-expectation activity, delivering high-quality and cost-effective outcomes in student learning [24]. However, most of the studies were conducted in senior-year students. A guideline is useful to direct the HFPS and help students learn more effectively. In current nursing education, HFPS is employed in various courses to enhance students’ understanding of patients’ conditions and related treatment and care. Early application of HFPS in junior students may help them develop more personal and professional skills and better learning attitudes. To address this, a modified HFPS guideline was designed based on the Healthcare Simulation Standards of Best Practice (HSSOBP) by the International Nursing Association for Clinical Simulation and Learning [INACSL] [25] to provide systematic approaches to learning tasks and ensure students perform as expected throughout the learning process [22]. Four major sessions from HSSOBP, namely pre-briefing, simulation design, facilitation, and debriefing, were adopted to design a modified HFPS guideline for this study. This study aimed to examine the modified HFPS guidelines’ impact on students’ satisfaction and confidence in HFPS learning, and early application in junior students may develop personal and professional skills and better learning attitudes. The results could triangulate the findings with students’ narratives after HFPS to understand how they achieved satisfaction and confidence in learning.

## 2. Materials and Methods

### 2.1. Study Design

A quasi-experimental with one intervention and one control group was conducted at a single tertiary institution in Hong Kong SAR, China between November 2021 and June 2022.

### 2.2. Study Objectives 

The objective aims to investigate the impact of the modified HFPS guideline on student satisfaction and self-confidence in learning. By comparing the modified guideline with the standard HFPS, the study intends to evaluate whether the modified guideline leads to higher levels of SSSCL among first-year nursing students. The objectives align with the overall purpose of the study, which is to assess the effectiveness of the modified HFPS guideline in improving student satisfaction and self-confidence in learning.

### 2.3. Study Setting and Sampling

Students aged ≥ 18 years were recruited. Those who had received HFPS training before or had experienced clinical placement were excluded to avoid contamination. The sample size was calculated to reach a desired power of 0.95 and a type I error of 0.05 with an effect size of 0.5 based on a past study [26] using G*Power 3.1.9.4. The calculated minimum required number of participants was 176 students (88 in each group).

### 2.4. Modified Guideline for HFPS as the Study Framework

The modified HFPS guideline was based on the HSSOBP [25], which was developed to guide the integration, use, and advancement of simulation-based experiences in academia, clinical practice, and research. The HSSOBP is a comprehensive and evidenced-based tool that includes inputs from multiple healthcare professionals and experts in simulations [25]. It consists of nine standards, of which four were used to design the structured guideline for this study, as they were deemed most applicable. These four HSSOBP standards, namely pre-briefing, simulation design, facilitation, and debriefing, provided a systematic approach to direct students in engaging in their learning and simulated activities. The pre-briefing consists of preparation and briefing to ensure that students had the necessary learning materials, understood the ground rules, and were aware of their roles and responsibilities in the HFPS. Students are required to understand specific learning outcomes before HFPS. The simulation design provided a structural framework to develop effective logistics and strategies (including simulation case design) for promoting learning goals and improving the quality of care and patient safety. Facilitation aimed to provide guidance to students to meet their learning needs and achieve learning outcomes. The facilitator is assumed to be responsible for managing the entire HFPS and providing support to students to work cohesively during their simulation experience. Debriefing is a process that includes feedback, clarification, and guided reflection. The debriefing is essential to help students identify their strengths and weaknesses, gaps in knowledge, skills, personal attitudes, and emotional management in a simulation clinical situation. To evaluate the effects of the modified HFPS guideline, two groups were assigned either intervention or standard treatment. The differences in the four HFPS sessions between the two groups are illustrated in Table S1. Students in the intervention group were provided with the modified HFPS guideline as the intervention, which involved a more systematic approach to enable students to learn through the four sessions in a 2-h HFPS. Conversely, those in the control group received standard treatment with basic instructions for HFPS over the same period. This indicated that the standard treatment provided basic information and support from the facilitator in the four HSSOBP standards.

### 2.5. Instruments

The Student Satisfaction and Self-Confidence in Learning (SSSCL), which was developed by the National League of Nursing [27], would be used in this study. It consists of 13 items with a 5-point Likert scale (1 = strongly disagree and 5 = strongly agree) to measure students’ perception of their satisfaction and self-confidence in learning. Five items are related to the subscale of students’ satisfaction (SS) in simulation-based learning activities, and the remaining eight concern the subscale of self-confidence in learning (SCL). The Cronbach’s alphas for the overall SSSCL and the subscales of SS and SCL were 0.95, 0.96, and 0.92, respectively, indicating excellent reliability in this study.

### 2.6. Study Procedure

Prospective participants were recruited via email and asked to select from three available timeslots for the HFPS. Students who agreed to participate were randomly assigned to either the intervention group, which received the HFPS following the new guidelines, or the control group, which received the standard guideline, according to their preference. Each laboratory group consisted of around eight to ten students, and the research assistant (RA) allocated students to the corresponding group. The RA was not involved in the implementation of HFPS. Once a group was filled, the RA contacted the students about the time and venue of the HFPS and emailed them the HFPS packages for preparation at an acceptable period, which was three days before HFPS for the students in the control group and one week for those in the intervention group. Two researchers were responsible for teaching the intervention and control groups, respectively, to ensure consistency. The tutorials were held at different campuses of the institution to avoid contamination. Students completed a baseline questionnaire before receiving the simulation on the study day and completed the same set of questionnaires immediately after the debriefing session.

On the day of HFPS, students were divided into three small groups and took turns in the role-play session to care for the simulated patient, with each group having 20 min in the simulation session. While one small group was assigned to the role-play session, the other two watched and provided feedback. In the debriefing session, students reflected on their learning throughout the HFPS, gave feedback to one another, and received feedback from the tutor. After the debriefing, students were asked to complete the post-intervention SSSCL survey and answer six open-ended questions about their learning in terms of satisfaction and confidence in learning through HFPS. The questions focused on the learning materials provided, the role-play session, the debriefing, and their effect on confidence in learning. The questions were: ‘What do you think about the learning materials provided before the HFPS?’, ‘What do you think about the effect of learning materials on your confidence in learning through HFPS?’, ‘What do you think about the role-play you performed in the HFPS?’, ‘What do you think your role-play in the HFPS will affect your confidence in learning?’, ‘What do you think about the debriefing after the HFPS?’, and ‘What do you think about the effect on your confidence in learning after the HFPS?’.

### 2.7. Data Analysis

Statistical analyses were performed by a data analyst who was blinded to the students’ allocation. Data were analysed using IBM SPSS version 26. Chi-square statistics were applied to compare the demographic characteristics (categorical data) between the intervention and control groups. Two-sample *t*-test statistics were applied to compare the student satisfaction and self-confidence in learning between the two groups. A two-sample independent *t*-test was used to examine the change of SSSCL between baseline and post-intervention (after debriefing) between the two groups. Secondary data analysis was conducted by ANOVA to examine the effect of HFPS on the change of SSSCL, adjusted for confounding factors. All statistical tests involved were two-sided, and *p*-values of <0.05 were considered statistically significant.

### 2.8. Ethical Consideration

Ethical approval was obtained from the research committee of the study institution (REC2021102). Informed consent was obtained from the students who agreed to participate. The participants were assigned by individual serial numbers, and the researchers would not be able to identify the participants during data analysis. All data with personal information were kept confidential.

## 3. Results

### 3.1. Students’ Characteristics

A total of 189 students were recruited in this study without attrition, with 92 students (48.7%) in the intervention groups and 97 (51.3%) students in the control groups. Table 1 shows the demographic characteristics and students’ satisfaction (SS) and self-confidence in learning (SCL) at baseline. Among the sampled participants, 73% participants were female, and the mean age was 20.56 (SD = 3.14). Around 71% of participants were studying for a bachelor’s degree, and the remaining 29.1% were studying higher diploma. Over half of the participants were in the first year of study (54%), and the remaining 46% were in the second year of study. The baseline demographic characteristics were similar between the intervention and control groups, except that a higher proportion of participants studying for a bachelor’s degree in the intervention group (79.3%) than in the control group (62.9%, *p* = 0.013). Both groups have similar levels of student satisfaction and self-confidence in learning at baseline.

### 3.2. Analysis of Outcomes

To assess the effectiveness of the intervention, the pre- and post-intervention scores of all subscales (SS and SCL), as well as the overall SSSCL, were compared between the intervention and control groups. The results showed a significant improvement in all subscales and overall SSSCL scores in both groups (<0.001). Table 2 illustrates the changes in subscales of SS and SCL and the overall SSSCL scores, which were observed to have improved in both intervention and control groups after the simulation.

Compared with the control group, participants who were in the intervention group recorded a higher improvement in SSSCL (mean change in the intervention group = 10.05 vs. 6.97 in the control group, *p* = 0.004), as well as both the SS and SCL scores (*p* = 0.004 and 0.025 respectively) (Table 2); all subscales were found to have significant differences between the two groups. Consistent results were observed after accounting for the confounding variable (Table 3).

### 3.3. Effects of the Guideline on SSSCL through HFPS

Most students reported feeling satisfied and confident in their learning at each stage, according to the narrative feedback from the six open-ended questions. Students in the intervention group reported higher levels of satisfaction and confidence in learning than those in the control group. Some students in the intervention group mentioned that they had more satisfaction and confidence in learning due to the learning engagement at each stage. They found that when they had more satisfaction, they had better confidence in learning. During the preparatory stage, students in the intervention group followed the guideline and read the learning materials to manage the simulated patient. They reflected that the materials were useful in enhancing their understanding of the health problem and related management. In the role-play session, students in the intervention group were able to manage the scenario more efficiently. During the debriefing, all students learned from the educator and group feedback and their own self-evaluation. Table S2 summarizes students’ feedback on their satisfaction and confidence in learning through three sessions of HFPS in the two groups.

## 4. Discussion

This study found significant improvement in the SSSCL in both groups, but there were more positive effects of the modified HFPS guideline on SSSCL through HFPS in the intervention group. All subscales of the SSSCL (SS and SCL) and the overall SSSCL showed significant differences (*p* < 0.001) between the pre- and post-intervention periods in both groups, indicating that HFPS itself improved student learning throughout the four-session HFPS [22]. HFPS uses advanced and innovative technology to foster student learning and learning motivation, providing a simulated clinical setting with a patient to allow students to actively participate in giving comfort care interventions, interacting with the patient, working with teammates, and receiving feedback from their facilitator [4]. Therefore, HFPS is an effective teaching method to allow students to practice patient care with learned knowledge and skills [18,21], aiming to enhance their clinical judgment and decision-making ability [8]. The modified guideline provided the necessary information and adequate support for students to be engaged in implementing care in a simulated patient situation during the process of HFPS, which informs more promising effects on student satisfaction and confidence in learning. Therefore, the HFPS guideline is a useful tool to enhance student learning and competence.

Comparing the changes in the subscales between the two groups, all subscales, particularly the subscales of SS (*p* = 0.004) and overall SSSCL (*p* = 0.004), showed significant differences before and after the intervention, with students in the intervention group reporting more changes in all SSSCL subscales. This suggests that the modified guideline greatly improved student learning, providing clearer direction and information for students to learn systematically, effectively, and sensibly [28]. The four sessions of HSSOBP were useful and effective in increasing students’ SSSCL from their own self-directed study, group collaboration, self-reflection, and evaluation or feedback from peers and the tutor. Importantly, students need to engage in the entire four-session HFPS to obtain the benefits of SSSCL improvement [25]. Throughout this learning process in HFPS, students had the opportunity to increase their satisfaction and self-confidence by acquiring new knowledge and skills, ultimately enhancing their competence in clinical judgment and management [17,21,29]. Therefore, the modified HFPS guideline provides clear instruction and learning support that motivates students to engage in HFPS and improve their SSSCL.

The narratives of students in the intervention group substantiated the quantitative results, demonstrating more satisfaction and confidence in learning throughout the HFPS learning process. They found the learning material useful in making clinical judgments more confidently. Students can achieve a better sense of accomplishment when they are appropriately directed to learn and prepare. They also develop critical assessment and management skills to better understand the patient’s experience and clinical practice in HFPS [30], ultimately enhancing their competence in clinical management [11,17]. During the role-playing session of the HFPS, students actively engaged in learning and practicing by interacting with the simulated patient and their teammates. They received opportunities to provide direct patient care and handle problem-based clinical situations, including sudden changes in health conditions, patient safety issues, and ethical concerns [21,31]. Working as a team in HFPS allowed students to collaborate with other team members for decision making and develop their personal and professional strengths together [14,15]. When students encountered difficult handling situations, they worked together for better clinical judgment and decision making [14,15,32]. Moreover, students in the intervention groups reported higher SSSCL through collaborative teamwork in the HFPS, which increased their competence in practicing safely and with appropriate intervention for the simulated patient. They also found that they learned from their educator, whose involvement as a facilitator enhanced their motivation and direction to learn more effectively during the role-play session of the HFPS [33].

In the debriefing session, all students appreciated the group and educator feedback, which allowed them to gain more learning and self-evaluate their performance for better practice and self-improvement [13,14,15,21]. Debriefing should be conducted as early as possible after the HFPS so that students can self-evaluate their performance for better practice and self-improvement [34]. In case immediate debriefing is not allowed, written self-debriefing is an alternative [34]. Despite a simulated situation, students are facing a range of emotions that profoundly stimulate students’ learning and performance. Debriefing is, therefore, also beneficial to reduce psychological burden and distress when they have a similar situation in the real clinical setting [24,34]. Importantly, the HFPS environment tolerates errors and allows students to improve their professional development [35]. While students are allowed to make mistakes in the HFPS, they are also reminded to be more alert when practicing in similar clinical situations in future real settings. Therefore, debriefing informs the success of appropriate clinical decisions and increases teaching quality [36].

This study successfully demonstrated the benefits of the modified HFPS guideline for student learning by increasing SSSCL through HFPS. Despite only a part of HSSOBP [25] being adopted in this study, the modified guideline, comprising four HFPS sessions: pre-briefing, simulation design, facilitation, and debriefing, allowed students to learn step by step. Figure S1 shows a conceptual framework for the association of these four sessions with student learning and their satisfaction and confidence in learning and how students learned at each session of HFPS. It is important to note that students’ self-study, their involvement, the tutor’s facilitation, feedback from peers and tutor, and students’ self-evaluation were also the key components to enhance their satisfaction and self-confidence in learning through HFPS. In general, the HFPS is usually employed in senior-year students to encourage them to practice their learned knowledge and skills. In this study, HFPS is also effective in stimulating students to learn individually and in a group, enhancing their learning attitudes, confidence, and satisfaction. Early development of confidence and satisfaction in learning ultimately allows students to enhance competence in practice, clinical judgment, and decision-making abilities. Thus, a structured guideline should be added to nursing courses with HFPS in the curriculum to facilitate students’ learning. The results also promote the awareness of nurse educators in designing guidelines for HFPS-related activities to enhance SSSCL in learning, which is crucial for clinical judgment and decision making.

### Strengths and Limitations

Strengths of this quasi-experimental with control study include providing reliable and accurate evidence of the effects of the modified guideline for HFPS. However, the generalizability of the results is limited due to the recruitment at a single professional training institution. Similar studies in multiple centers should be conducted to increase generalizability. The absence of randomization can limit the researcher’s ability to make strong causal claims about the intervention’s effectiveness and may introduce selection bias, as there may be systematic differences between the groups being compared.

## 5. Conclusions

HFPS has recently emerged as an effective teaching and learning method in professional training. The modified guideline in this study provides clear direction for students, including junior students, to learn step-by-step and apply specific knowledge and skills to a patient with specific health needs in a simulated clinical setting. The HFPS guideline improves students’ ability to make informed clinical judgments and effective decisions, leading to enhanced patient care. The students’ narratives supported the findings of the quantitative results on SSSCL through HFPS. A conceptual framework (Figure S1) was developed to understand student learning, their satisfaction, and confidence in learning, as well as the ultimate learning outcomes through HFPS. Throughout the learning process with the structured HFPS guideline, students can learn more effectively with higher satisfaction and confidence in learning. The results of this study increase educators’ awareness of the application of an HFPS guideline in the training curriculum to achieve better teaching and learning outcomes.

## Figures and Tables

**Table 1:** Students’ characteristics, students’ satisfaction, and self-confidence in learning.

	Overall (n = 189)		Intervention (n = 92)		Control (n = 97)		*p* -Value
	n	%	n	%	n	%	(between Groups)
**Gender**							0.210
Male	51	27	21	22.8	30	30.9	
Female	138	73	71	77.2	67	69.1	
**Age**							0.027 *
mean age (SD)	20.56 (3.14)		21.04 (3.65)		20.04 (2.41)		
18–24	170	89.9	85	92.3	85	87.6	
25 or older	19	10.1	7	7.7	12	12.4	
**Program**							0.013 *
Higher Diploma	55	29.1	19	20.7	36	37.1	
Bachelor of Science	134	70.9	73	79.3	61	62.9	
**Study Year**							0.919
1	102	54.0	50	54.3	52	53.6	
2	87	46.0	42	45.7	45	46.4	
**Student satisfaction and self-confidence in learning**	Mean	SD	Mean	SD	Mean	SD	*p*-value
SS	18.25	3.40	17.83	3.17	18.64	3.61	0.097
SCL	28.78	3.77	28.59	3.97	28.96	3.57	0.543
Overall SSSCL	47.03	6.98	46.41	6.57	47.61	7.35	0.239

SS: Student Satisfaction; SCL: Self-Confidence in Learning; SSSCL: Student Satisfaction and Self-Confidence in Learning. * *p* < 0.05.

**Table 2:** Comparison of the changes in students’ satisfaction and self-confidence in learning before and after HFPS between intervention and control groups.

	Pre- and Post-Change			
	Mean	SD	*p*	95% CI
SS				
Intervention	5.14	3.27	0.004 **	−2.67 to −0.50
Control	3.56	4.18		
SCL				
Intervention	4.91	3.85	0.025 *	−2.81 to −0.19
Control	3.41	5.18		
Overall SSSCL				
Intervention	10.05	6.32	0.004 **	−5.18 to −0.99
Control	6.97	8.17		

SS: Student Satisfaction; SCL: Self-Confidence in Learning; SSSCL: Student Satisfaction and Self-Confidence in Learning. * *p* < 0.05; ** *p* < 0.01.

**Table 3:** Intervention effect on the changes of students’ satisfaction and self-confidence in learning.

	Mean	SE	*p*
SS			
- Treatment (Intervention)	4.97	3.32	0.004 *
- Program (Bachelor)	9.73	6.20	0.312
SCL			
- Treatment (Intervention)	4.75	3.64	0.035 *
- Program (Bachelor)	4.75	3.64	0.304
Overall SSSCL			
- Treatment (Intervention)	9.73	6.20	0.005 *
- Program (Bachelor)	4.97	3.32	0.473

SS: Student Satisfaction, SCL: Self-Confidence in Learning, SSSCL: Student Satisfaction and Self-Confidence in Learning. * *p* < 0.05.

## Data Availability

The data presented in this study are available on request from the corresponding author. The data are not publicly available due to keep the confidentiality.

## Supplementary Materials

The following supporting information can be downloaded at: https://www.mdpi.com/article/10.3390/nursrep13030090/s1, Figure S1: A conceptual framework on the association of these four sessions with student learning and their satisfaction and confidence in learning; Table S1: Differences of the four sessions in HFPS between the two groups; Table S2: The summary of students’ feedback on their satisfaction and confidence in learning through three sessions of HFPS in the two groups.

## Public Involvement Statement

Guidance for Reporting Involvement of Patients and the Public Long Checklist was completed.

## Guidelines and Standards Statement

This manuscript was drafted against the CONSORT Extension Checklist [37] for A mixed (a quasi-experimental and qualitative study) research.
