# Supplementary material for: A Modified Guideline for High-Fidelity Patient Simulation to Improve Student Satisfaction and Self-Confidence in Learning: A Mixed Study
Source: Nurs Rep. 2023 Jul 28;13(3):1030–9. doi: 10.3390/nursrep13030090 (PMC10443364; doi:10.3390/nursrep13030090)
Supplement: Supplementary file 1 [file nursrep-13-00090-s001.zip › nursrep-2519867-supplementary.pdf]

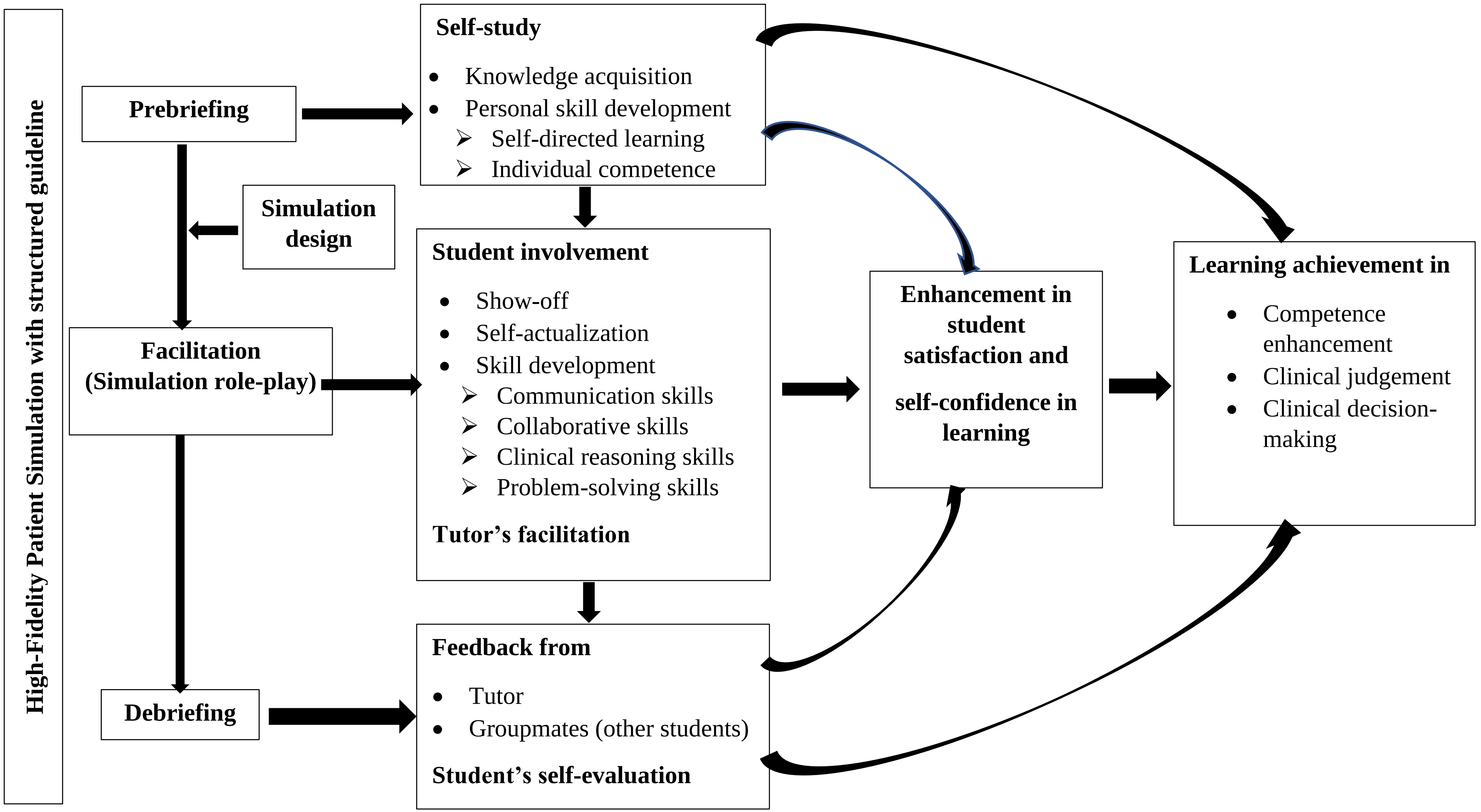

Figure S1. A conceptual framework of five components for student learning in terms of student satisfaction and confidence in learning in high-fidelity patient simulation

Table S1. Differences of the four sessions in HFPS between the two groups

Sessions	Characteristics	Standard HFPS<br>*The educator was responsible for the students in the control groups.	HFPS with modified guideline<br>*The educator was responsible for the students in the intervention groups.
Pre-briefing	Pre-simulation study	Students received basic information for HFPS<br><br>• General lab and simulation rules and regulations.<br>• Simulated case scenario with brief information provided by the educator.<br>• Orientation of the simulation environment was given by the educator*<br>• The materials were sent at least three days before HFPS.	Students will receive HFPS guidelines at least one week before HFPS, including the information below.<br>• Ground rules and regulations of the lab and simulation<br>• Expected roles and responsibilities of students and student groups.<br>• The expected learning outcomes<br>• The case scenario information and condition in 3 stages of the patient condition (for 3 individual small groups)<br>• Learning materials and activities regarding the health problem of the simulated patient were sent to students.<br>• Students were encouraged to have more self-study.<br>• Orientation of the simulation case and environment was given by the educator.<br>• The educator gave an introduction to the logistics of the HFPS.
Simulation design	Development of HFPS case scenario, ground rules, and regulations, intended learning objectives, logistics, and resources/equipment	• A case scenario with a health problem (cardiac problem), was the same case for the intervention group.<br>• Designed based on the learning objectives only.<br>• The educator who was the facilitator in the control group had more than 20 years of experience in clinical practice in acute care and education.<br>• Lab technicians or a nurse controlled the simulation for the control group.	• The guideline was based on the HSSOBP<br>• A case scenario with a health problem (cardiac problem) was the same case for the control group.<br>• Designed based on the learning objectives.<br>• The educator who was also the facilitator in the intervention group had more than 20 years of experience in cardiac clinical practice and education.<br>• A clinical expert was consulted for updated evidence-based practice.<br>• An experienced simulationist was consulted and involved in the simulation for the intervention group.

Facilitation	Role play in HFPS	• Students were divided into 3 small groups and expected to perform nursing care and skill practice in the simulated case.<br>• Students were expected to apply their learned knowledge and skills.<br>• Duration: 20 minutes<br>• Students were required to self-manage the simulated situation with limited facilitation by the educator.	• Students were divided into 3 small groups and expected to perform nursing care and skill practice in the simulated case.<br>• Students were expected to apply their learned knowledge and skills.<br>• Duration: 20 minutes<br>• Students were facilitated by the educator during their role-playing in the simulation.<br>• Students who were observers are required to record the areas in which the players had been well performed and that could be improved regarding their observation of the students who were role-playing in HFPS.<br>• Students had some guiding questions to direct them to observe the performance of the players.
Debriefing	Debriefing	Mainly students had feedback from the educators.	• All students were required to give comments on the performance of the other groups through self-/group-reflection, open discussions, and critical thinking using an integration of knowledge and skill application.<br>• Individual students were requested to reflect on their performance and identify solutions.<br>• The educator gave comments on overall individual students and group performance for improvement.

Table S2. The summary of students' feedback on their satisfaction and confidence in learning through three sessions of HFPS in the two groups.

Groups	Outcome measures	Pre-briefing session	Direct response from students	Facilitation (Simulation role-play)	Direct response from students	Debriefing	Direct response from students
Control group	Student satisfaction	All students read the case scenario and felt satisfied with being informed about the health problem of the patient simulation.	Student 158, "I feel satisfied to understand the case before the simulation. I knew it was related to a cardiac patient."<br><br>Student 65, "I did not know much about the simulation, but the case scenario prepared me better.<br>The information. I do not know all of it, e.g., the checklist for thrombolytic therapy. Perhaps, I will after the simulation"	Students faced difficulties in reacting to the patient's needs.	Student 129, "I didn't know what to do in the simulation. It's really hard to know what I should do first and what next. I felt helpless and realized that I needed to prepare better for clinical practice."	All students felt satisfied with learning more about themselves and the areas they should improve.	Student 48, "I have learned a lot in the debriefing. The tutor's feedback let me understand the appropriate response and care for the patient. I haven't had clinical experience before. It's good to have debriefing so that we can evaluate ourselves and understand how to improve our practice."
	Confidence in learning	Most of the students expressed fair confidence in learning.	Student 153, "Nothing very special in terms of confidence because I do not know much about simulation."	Most of the students expressed helplessness and frustration because of uncertainties in reacting to the patient's needs but they learned that knowledge and skills were	Student 119, "I realized that I was incompetent in providing care for the patient. I was not sure if the care I provided was appropriate. It just came out of my common sense. I learned a lot during the simulation (role-playing session), but I should have been even better prepared to be more	All students admitted that the debriefing increased their confidence in learning as they understood more about their weaknesses	Student 118, "After the simulation, the tutor commented on our performance for our improvement. The feedback was very valuable to enhancing my understanding of the case problem and its management."

				essential and that they needed to be prepared before caring for patients.	confident in giving patient care.”	and the areas they could improve.	
Intervention group	Student satisfaction	All students read the case scenario and the provided learning materials. Some students were satisfied with the learning through further individual study when they had problems and searched for related information through other channels to understand more about the case.	Student 99, “I appreciated learning through reading the provided materials and surfing different websites for additional information. I now understand more about health problems and their management.”	Most of the students expressed that they were satisfied with the performance in the simulation session. Some of them explained that the learning materials enhanced their understanding of the health problem and related management. Their confidence was improved accordingly.	Student 55, “The simulation was useful for me in applying the knowledge and skills. The learning materials gave me important information to critically think about the appropriate patient care.”<br><br>Student 61, “The simulation can let me taste the role of a nurse to provide care to the patient. I felt anxious at the beginning, but I learned from the simulation and teamwork.”	All students expressed that they had learned a lot in the debriefing and felt satisfied with their learning. Some students expressed that their confidence was increased after debriefing because they felt being guided and encouraged to be improved.	Student 97, “Simulation is like the real clinical situation. I learned a lot from the tutor’s feedback. I learn about my limitations and I can improve myself accordingly.”<br><br>Student 87, “I appreciate the comments from the tutor. I am satisfied with the learning in this session (debriefing) because I can evaluate myself and know how to perform better.”
	Confidence in learning	All students expressed to have more confidence after reading the materials.	Student 74, “The provided learning materials were useful to boosting my confidence and competence in caring	In the group, all students learned more effectively. They collaborated and	Student 40, “It is important to know what to do next. The simulation tested how much I had learned and how to apply them. I realize that	In the debriefing, students received feedback from their	Student 149, “The debriefing is important for me to understand more about myself. The feedback from the

		They found methods to prepare themselves better before the HFPS. When they learned more, they were more willing to be involved in the simulation to give care to the patient.	for the patient in the simulation."	prioritised the care. They had more confidence in providing care for the client.	adequate preparation is important for good practice because I was required to read the material before the simulation, which helped me deliver care better in the simulation.<br><br>Student 171, "Teamwork is important in the simulation. When we worked together, I felt the support from my group mates and was more confident in what I was doing."	educators and teammates. Most of the students expressed that they realised their strengths and weaknesses for their improvement. They also gained more knowledge and clinical experience from their educators to enhance their competence and confidence in further learning and clinical practice.	educator and teammates was very useful. The educator gave us suggestions to correct and improve our practice. Our competence in clinical practice must be backed up by adequate confidence in practising patient care at the bedside."
